# Influence of segmental supply of Cilioretinal artery on morphology of diabetic macular edema

**DOI:** 10.1186/s12886-021-01812-x

**Published:** 2021-01-21

**Authors:** Rehana Khan, Mahesh Shanmugam, Rajesh Ramanjulu, Jay Chablani, Niharika Singh, Avadhesh Oli, Rajiv Raman

**Affiliations:** 1 Shri Bhagwan Mahavir Vitreoretinal Services, Sankara Nethralaya, 18 College Road, Sankara Nethralaya, Chennai, Tamil Nadu 600 006 India; 2 Department of Vitreoretinal services and Ocular Oncology Services, Sankara Eye Hospital, Bengaluru, Karnataka India; 3 Srimati Kannuri Santhamma Centre for Vitreo-Retinal Diseases, L. V. Prasad Eye Institute, Hyderabad, Telangana India

**Keywords:** Cilioretinal artery, Center involving macular edema, Non-center involving macular edema, Segmental supply, Optical coherence tomography, Retinal thickness, ETDRS

## Abstract

**Background:**

The supply of Cilioretinal artery (CRA) to different layers of the retina influences retinal pathologies such as diabetic retinopathy (DR). Since the supply of CRA is segmental, our aim was to analyze the location of CRA with respect to non – center involving diabetic macular edema (DME) differentiated by various segments and center involving DME based on Early Treatment of Diabetic Retinopathy Study (ETDRS) scale using optical coherence tomography (OCT).

**Methods:**

A retrospective study was conducted in which forty-three patients with various stages of DR and the presence of CRA were identified. Presence and location of CRA was recognized using fundus fluorescein angiography. Classification of DME was based on ETDRS subfields on OCT.

**Results:**

Evaluation of 26 men and 17 women with varying degrees of severity involving DR revealed the presence of unilateral CRA in 40 subjects and bilateral CRA in 3 subjects. When CRA supplied the central area, maximum retinal thickness was noted at the temporal quadrant (271.67 ± 164.02 μm) along with non - center involving DME (194.87 ± 121.06 μm); when CRA supplied the lower area, maximum retinal thickness was noted at the superior quadrant (293.64 ± 159.36 μm) along with center involving DME (395 ± 285.75 μm) and when it supplied the upper area, maximum retinal thickness was noted at the nasal quadrant (293.49 ± 176.18 μm) along with center involving DME (292 ± 192.79 μm).

**Conclusion:**

The presence of CRA seems to influence the morphology of the retina amongst patients diagnosed with DR by altering the segments involved in DME based on its supply location. However, further studies with a larger sample size are warranted to strenghten this association.

## Background

The outer retina is supplied by choroidal circulation whereas the inner retina by retinal circulation. There are anatomical, hemodynamic, and autoregulatory differences in these circulations. The changes in retinal circulation are well characterized in diabetic retinopathy (DR) based on previous literature, however, changes in choroidal circulation are not. A recent study by Kim et al. [1], identified the choroidal vascular changes in diabetic patients by measuring the choroidal vascularity index (CVI). The authors hypothesized that a reduction in the choroidal vascular component was apparently noted in patients with type-2 diabetes mellitus (DM), even when there was no evidence of DR.

Cilioretinal artery (CRA) originates from the ophthalmic artery and fills up during choroidal flow of fundus fluorescein angiography (FFA). The difference in the supply to different layers of the retina by choroidal and retinal circulation may influence the phenotype of retinal pathologies such as DR.

Recent studies have hypothesized that the vascular structures or substances from the choroid might influence diabetic maculopathy [2]. Knudsen and Lervang showed that CRA was detected in the eye amongst diabetic patients who have either asymmetric bilateral diabetic maculopathy or more severe maculopathy including clinically significant macular edema [3]. A similar study conducted by Landa et al., using a retinal function imager, also showed an increased occurrence of diabetic macular edema (DME) in association with the presence of CRA in diabetic eyes [2].

However, usually, the supply of CRA is segmental. The effect of this segmental choroidal supply based on location in DR has not been evaluated. In this study, our focus was to analyze the location of CRA with respect to non – center involving diabetic DME differentiated by various segments and center involving DME based on Early Treatment of Diabetic Retinopathy Study (ETDRS) scale using optical coherence tomography (OCT).

## Methods

FFA databases from three tertiary care centers in south India were utilized to identify a sample population of patients with DM along with the presence of CRA. On screening the databases from 2015 to 2017, a total of 43 (26 men and 17 women) patients were identified with various stages of DR and the presence of CRA. Among these 43 patients, unilateral CRA was present in 40 patients and bilateral CRA in 3 patients. In patients with unilateral CRA the affected eye, as well as in patients with bilateral CRA, the right eye were taken as the CRA present eyes (Case group, *N* = 43). In patients with unilateral CRA, the fellow eyes were taken as the CRA absent group (Control group, *N* = 40). All patients in the study group had adult-onset DM with mild-to-severe non - proliferative diabetic retinopathy (NPDR) and proliferative diabetic retinopathy (PDR). Patients with neovascularization of the disc (NVD) and small fine CRA at the disc margin on angiogram were excluded.

### Clinical assessment

Baseline patient characteristics involving demographic details and medical history including duration of DM, associated systemic hypertension, treatment received including LASER photocoagulation, intravitreal injections and best corrected visual acuity were noted. Fundus photographs were graded for the presence of DR using ETDRS classification. Classification of DME was based on ETDRS subfields on OCT. [4]

FFA-proven CRA location presence and prominent regions of the macula (upper, central, and lower) being supplied by it were noted. OCT images were taken in Cirrus SD-OCT and the findings were entered carefully as center involving DME, non – center involving DME and the absence of DME. Possible presence of CRA in the other eye was also noted. The study was approved by the Institutional Review Board (Ethics committee), Vision Research Foundation and written informed consent was obtained from the subjects as per the Declaration of Helsinki.

#### Grading of DME

The grading of DME was done as follows [4]:

##### Center involving DME

Patients were diagnosed with center involving DME if on clinical examination, definite retinal thickening due to DME involving the center of the macula was observed. The spectral-domain optical coherence tomography (SD-OCT) showed loss of foveal contour, cystic space involving center of fovea, and neurosensory detachment involving the center of fovea. Central subfield thickness on OCT > 290 μm for women, > 305 μm for men was observed on SD-OCT (Cirrus; Zeiss).

##### Non – center involving DME

Patients were diagnosed with Non-center involving DME if on SD-OCT, definite retinal thickening due to DME within 3000 μm of the center of the macula but not involving the center of the macula was observed. The SD-OCT showed cystic spaces and or retinal thickening in non - central macular subfields.

##### No DME

Patients were diagnosed with no DME if they had normal central subfield thickness on OCT 209 ± 18 μm in men and 194 ± 23 μm in women.

#### Mean retinal thickness (in microns) according to early-treatment diabetic retinopathy study (ETDRS) protocol [5]

Clinically significant macular edema was considered if the retinal thickening or hard exudates were observed within 500 ± 50 μm of the center of the foveal avascular zone or zones of retinal thickening 1 disc area or larger, any part of which was within 1 disc diameter of the center of the macula [6]. Hence the identification of DME was based on both specific OCT features and retinal thickness.

#### Statistical analysis

SPSS (version 21·0) was used for statistical analysis. Descriptive statistics with mean and standard deviation values was performed for continuous variables after confirming normal distribution. Categorical data were presented as count data along with percentage values. Patient count, proportion and independent sample t-test were used to describe and compare DME status and the presence or absence of CRA with respect to disease severity in DR. Chi square test was used to differentiate the distribution of DME among patients with or without the presence of CRA. Anova test was performed to compare mean difference in area of retinal thickness for each quadrant based on location of CRA supply.

## Results

FFA evaluation of these 43 subjects with DM revealed a unilateral CRA in 40 subjects and bilateral in 3 subjects. (Table 1) shows the baseline characteristics of the study patients. The distribution of DR were well balanced between the CRA present and the CRA absent eyes.

**Table 1:** Baseline characteristics of the study patients

Variables	
Total number of patients	43
Unilateral CRA* patients, (N)	40
Bilateral CRA* patients, (N)	3
Eyes with CRA,* (N)	43
Eyes without CRA,* (N)	40
Age, (mean ± SD), years	57.66 ± 7.99
Duration of diabetes, (mean ± SD), years	11.52 ± 6.51
Men, N (%)	26 (60.47)
**Eye Distribution of DR**# with CRA* **(Cases), N (%)**	
Mild NPDR†	5 (11.63)
Moderate NPDR†	20 (46.51)
Severe NPDR†	12 (27.91)
PDR‡	6 (13.95)
**Eye Distribution of DR**# without CRA* **(Controls), N (%)**	
Mild NPDR†	6 (15)
Moderate NPDR†	18 (45)
Severe NPDR†	9 (22.5)
PDR‡	7 (17.5)

*CRA: Cilioretinal artery; #DR: Diabetic Retinopathy; †NPDR: Non - proliferative diabetic retinopathy; ‡PDR – Proliferative diabetic retinopathy

(Table 2) shows relationship of DME with area of supply by CRA. The subjects with center involving DME had more number of central area of supply by CRA and equal number of upper and lower CRA supply. The subjects with non-center involving DME and no DME had more of central supply and less of upper supply by CRA.

**Table 2:** Relationship of diabetic macular edema and area of supply by cilioretinal artery

Area of Supply by CRA*	Upper	Central	Lower
Center involving DME†, N(%)	5 (31.25%)	6 (37.5%)	5 (31.25%)
Non-center involving DME†, N(%)	2 (12.5%)	8 (50%)	6 (37.5%)
No DME†, N(%)	1 (9.09%)	6 (56.54%)	4 (36.36%)
*p*	*0.25*	*0.10*	*0.36*

*CRA: Cilioretinal artery; #DME: Diabetic macular edema

(Table 3) shows the distribution of stages of DR in DME with the presence and absence of CRA. The stages of DR were distributed in balance in CRA present and CRA absent group though not statistically significant. The distribution of non-center involving DME in the presence of CRA is comparatively more than in the absence of CRA. The distribution of center-involving DME and no DME in the presence of CRA is lower than in the absence of CRA. Statistical significance was noted in non – center involving DME group. The overall distribution of center and non – center involving DME was observed more in eyes with the presence of CRA (*p* = 0.062), whereas no DME was observed more in eyes without CRA (i.e control group).

**Table 3:** Distribution of stages of diabetic retinopathy in diabetic macular edema with presence and absence of cilioretinal artery

Diabetic Retinopathy and stages	Center involving DME**			Non - Center involving DME**			No DME*		
	CRA* present (***N*** = 16)	CRA* Absent (***N*** = 19)	***p***	CRA* present (N = 16)	CRA* Absent (***N*** = 7)	***p***	CRA* present (***N*** = 11)	CRA* Absent (***N*** = 14)	***p***
Any DR#, N(%)	16 (37.21)	19 (47.50)	*0.345*	16 (37.21)	7 (17.5)	***0.043***	11 (25.58)	14 (35)	*0.352*
Mild NPDR†, N(%)	2 (12.5)	3 (15.79))	*0.784*	1 (6.25)	0 (0.00)	*0.508*	2 (18.18)	3 (21.43)	*0.843*
Moderate NPDR†, N(%)	7 (43.75)	8 (42.11)	*0.923*	10 (62.5)	6 (85.71)	*0.276*	3 (27.27)	4 (28.57)	*0.943*
Severe NPDR†, N(%)	4 (25)	4 (21.05)	*0.784*	4 (25)	1 (14.29)	*0.575*	4 (36.36)	4 (28.57)	*0.684*
PDR‡, N(%)	3 (18.75)	4 (21.05)	*0.867*	1 (6.25)	0 (0.00)	*0.508*	2 (18.18)	3 (21.43)	*0.843*

**Fig. 1:**
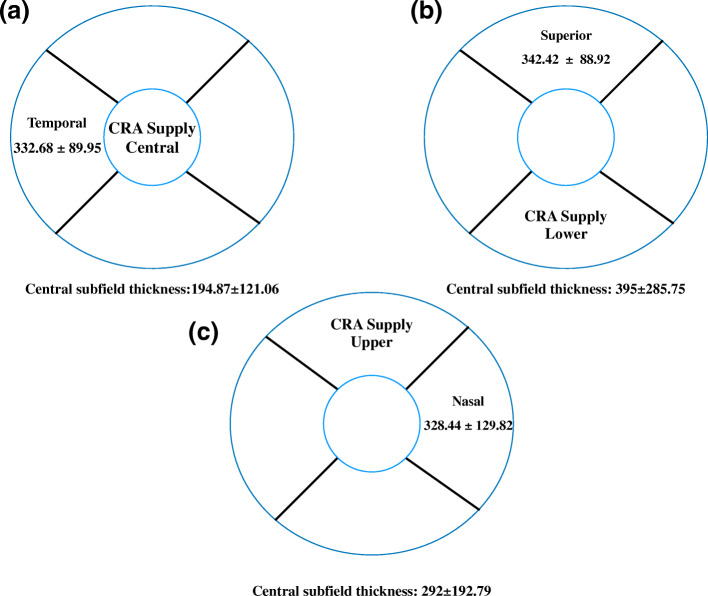
Supply of cilioretinal artery and maximum retinal thickness in ETDRS subfields on OCT. (a) In central CRA supply, the maximum retinal thickness was noted at the temporal quadrant (332.68 ± 89.95) and has non - center involving DME (Central subfield thickness:194.87 ± 121.06 μm). (b) In lower CRA supply, the maximum retinal thickness was noted at the superior quadrant (342.42 ± 88.92) and has center involving DME (Central subfield thickness: 395 ± 285.75 μm). (c) In upper CRA supply, the maximum retinal thickness was noted at the nasal quadrant (328.44 ± 129.82) with center involving DME (Central subfield thickness: 292 ± 192.79 μm)

*CRA: Cilioretinal artery; #DR: Diabetic Retinopathy; †NPDR: Non - proliferative diabetic retinopathy; ‡PDR – Proliferative diabetic retinopathy; **DME: Diabetic macular edema

(Table 4) shows the relationship between area of supply of CRA and area of retinal thickness. (Fig. 1) shows area of supply of CRA and maximum retinal thickness in ETDRS subfields on OCT. When CRA supplies the central area, the maximum retinal thickness was noted at.

**Table 4:** Relationship between area of supply of cilioretinal artery and area of retinal thickness

Area of supply of CRA*	Superior	Temporal	Inferior	Nasal	Central subfield
Center, (mean ± SD)	324.34 ± 58.83	332.68 ± 89.95^#^	320.84 ± 83.26	328.42 ± 77.57	194.87 ± 121.06
Upper, (mean ± SD)	293.5 ± 132.15	308.69 ± 160.56	325.13 ± 129.40	328.44 ± 129.82^#^	292 ± 192.79
Lower, (mean ± SD)	342.42 ± 88.92^#^	332.04 ± 101.38	319.08 ± 112.80	336.62 ± 127.90	395 ± 285.75
*p*	0.441	0.858	0.991	0.972	0.962

*CRA: Cilioretinal artery; ^#^*Maximum retinal thickness*

the temporal quadrant and had non-center involving DME, when CRA supplies the lower area, the maximum retinal thickness was noted at the superior quadrant and had center-involving DME and with CRA supply in the upper area, the maximum retinal thickness was noted at the nasal quadrant with center-involving DME.

## Discussion

The incidence of CRA involves varied reporting among previously published studies. A study done by K S Mehra et al. [7], reported an overall incidence of 6.9% within the Indian population, however, Lorentzen et al., Lei Liu et al. and Collier et al. reported a much higher overall incidence of 26, 35 and 21.6% respectively [8, 9]. Additionally, a study published by Jain I S et al., reported a 22.8% incidence of CRA in the Chandigarh (city in North India) population [10]. Hence the incidence of CRA varies between 6 and 40%.

In this study, we evaluated the association between the presence of CRA and varied morphology of DME. It was found that the patients with CRA had more non-center involving DME compared to center-involving DME, whereas the opposite findings were noted in patients without CRA. However, this difference was not statistically significant. We also observed that if the CRA supplied the central areas, the maximum retinal thickness was noted in the non-central area (temporally). It was also found that the presence of CRA alters the morphology of macular edema. This finding could be explained likely due to its presence being a protective factor to the area supplied by it from developing macular edema. This is in contrast to the findings reported in the study by Landa et al., which noted higher rates of DME in such eyes [2]. However, the authors did not classify the edema as center or non-center involving. As the histological structure of CRA is similar to the branches of the central retinal artery, it is assumed that it undergoes similar levels of hypoxic damage, or even greater due to the absence of “auto regulatory” features, which are a hallmark of retinal circulation [11]. However, CRA has a high flow velocity, which may compensate for the reduction of flow due to structural changes in choroid due to DM.

Recently, Ebraheem et al., had reported that the presence of a CRA results in decreased levels of sub retinal fluid (SRF) in treatment-naive nAMD patients compared to controls who lacked a CRA [12]. The authors concluded that CRA alters the haemodynamics of blood flow by diverting blood from the choroid to the inner retinal circulation, thereby resulting in an increase in the occurrence of CME and a decreased occurrence of SRF. A similar mechanism might be involved in the disparity noted regarding the altered distribution of DME among patients with or without CRA.

The strength of the study was that only patients with FFA-proven CRA were included. However, this reduced the sample size in each group, as those individuals with clinical suspicion but no proven presence of CRA on FFA were excluded. Systemic factors such as glycemic control, serum lipids, and blood pressure were not considered in our analysis as data for these variables were not available for all patients. In some cases, patients with non-center DME or no DME can progress into center involving DME, however better understanding of this progression requires future studies along with longitudinal analysis of these patients which is beyond the scope of our retrospective observational study.

## Conclusion

In summary, the presence of CRA seems to be correlated with an alteration in the morphology of DME. The segmental distribution of CRA is also noted to influence the segment involved in DME. However, further prospective studies with larger sample sizes are warranted to solidify this association.

## Data Availability

The datasets generated during and/or analysed during the current study are not publicly available, as it is against the organization/hospital (Vision Research Foundation, Chennai) policy. All anonymized data available upon request (nanu@snmail.org) and are being stored in Vision Research Foundation office.

## Abbreviations

AMD: Age-Related Macular Degeneration
CRA: Cilioretinal Artery
CME: Cystoid Macular Edema
CVI: Choroidal Vascularity Index
DM: Diabetes Mellitus
DR: Diabetic Retinopathy
DME: Diabetic Macular Edema
ETDRS: Early Treatment of Diabetic Retinopathy Study
FFA: Fundus Fluorescein Angiography
NPDR: Non-Proliferative Diabetic Retinopathy
NVD: Neovascularization of the disc
OCT: Optical Coherence Tomography
PDR: Proliferative Diabetic Retinopathy
SD-OCT: Spectral Domain-Optical Coherence Tomography
SRF: Sub Retinal Fluid

## References

[1] Kim M, Ha MJ, Choi SY, Park YH (2018). Choroidal vascularity index in type-2 diabetes analyzed by swept-source optical coherence tomography. Sci Rep.

[2] Landa G, Amde W, Haileselassie Y, Rosen RB (2011). Cilioretinal arteries in diabetic eyes are associated with increased retinal blood flow velocity and occurrence of diabetic macular edema. Retina..

[3] Knudsen LL, Lervang HH (2002). Can a cilio-retinal artery influence diabetic maculopathy?. Br J Ophthalmol.

[4] Raman R, Bhende M (2015). Diabetic Macular Edema. Medical & Vision Research Foundations.

[5] Sabouri MR, Kazemnezhad E, Hafezi V. Assessment of macular thickness in healthy eyes using cirrus HD-OCT: a cross-sectional study. Medical hypothesis, discovery and innovation in ophthalmology. 2016;5(3):104. PMC5347212 28293656

[6] Sánchez-Tocino H, Alvarez-Vidal A, Maldonado MJ, Moreno-Montañés J, García-Layana A (2002). Retinal thickness study with optical coherence tomography in patients with diabetes. Invest Ophthalmol Vis Sci.

[7] Mehra KS (1965). Incidence of cilio-retinal artery in Indians. Br J Ophthalmol.

[8] Lorentzen SE (1970). Incidence of cilioretinal arteries. Acta Ophthalmol.

[9] Liu L, Liu LM, Chen L (2011). Incidence of cilioretinal arteries in Chinese Han population. International journal of ophthalmology.

[10] Jain IS, Singh K, Nagpal KC (1972). Vessels at the disc margin (cilioretinal and other simulating cilioretinal vessels). Indian J Ophthalmol.

[11] Giuffre G, Montalto FP, Amodei G (1987). Development of an isolated retinal macroaneurysm of the cilioretinal artery. Br J Ophthalmol.

[12] Ebraheem A, Uji A, Abdelfattah NS, Nittala MG, Sadda S, Le PV (2018). Relationship between the presence of a Cilioretinal artery and subretinal fluid in Neovascular age-related macular degeneration. Ophthalmology Retina.

